# *MGAT1* knockout in human dendritic cells enhance CD8^+^ T cell activation

**DOI:** 10.3389/fimmu.2025.1588795

**Published:** 2025-12-17

**Authors:** Anne Louise Blomberg, Betina Lyngfeldt Henriksen, Weihua Tian, Kerstin Skovgaard, Sarah Line Skovbakke, Steffen Goletz

**Affiliations:** 1 Biotherapeutic Glycoengineering and Immunology, Section for Medical Biotechnology, Department of Biotechnology and Biomedicine, Technical University of Denmark, Kgs Lyngby, Denmark; 2 Antiviral Immunomics, Section for Medical Biotechnology, Department of Biotechnology and Biomedicine, Technical University of Denmark, Kgs Lyngby, Denmark

**Keywords:** CD8+ T cell response, CRISPR/Cas9, DC differentiation, dendric cells (DCs), N-glycosylation, Glycoengineering

## Abstract

Dendritic cells (DCs) are crucial in regulating immune responses, making them a compelling target for immunotherapy. While DC vaccines have demonstrated safety and feasibility, their limited clinical efficacy underscores the need for strategies to enhance DC functionality. Emerging evidence highlights the regulatory roles of sialoglycans in DC biology, yet the structure-function relationships of other glycans remain poorly understood. To aid the understanding of DC glycobiology, we recently developed and validated a human model system based on genetically glycoengineered MUTZ-3-derived DCs and showed that ST6GAL1-mediated α2,6-sialylation specifically modulates CD4^+^ T cell activation. In this study, we knocked-out (KO) mannosyl (α-1,3-)-glycoprotein β-1,2-N-acetylglucosaminyltransferase (*MGAT1*) to investigate how the shift from complex to oligomannose N-glycans affects DC biology and function. *MGAT1* KO completely abolished the synthesis of complex and hybrid N-glycans. Differentiation of *MGAT1* KO MUTZ-3 cells into immature DCs (iDCs) induced upregulation of DC markers including CD1a, CD80, CD86, CCR6, and CD209, comparable to the upregulation observed in WT iDCs. Interestingly, *MGAT1* KO iDCs displayed an enhanced immunostimulatory profile, marked by increased surface densities of CD40, HLA-ABC, and HLA-DR, in combination with elevated mRNA levels of *NFKB1* and *IFNB1*. Consistent with this profile, *MGAT1* KO iDCs highly enhanced the activation and proliferation of allogeneic human CD8^+^ T cells *in vitro*, resulting in significantly higher levels of secreted proinflammatory cytokines compared to WT iDCs. This enhanced CD8^+^ T cell activation persisted under PD-L1 blockade, underscoring the robustness of the *MGAT1* KO–driven effect. Significantly elevated *NFKB1* levels in the *MGAT1* KO iDCs suggest enhanced NF-κB activity driving HLA and costimulatory molecule upregulation and robust CD8^+^ T cell activation. We further demonstrate that *MGAT1* KO promotes accelerated DC differentiation, yielding DCs that after three days of differentiation acquire the capacity to activate T cells. Building on previous research into sialic acids in DC biology, our findings reveal a regulatory role for complex and hybrid N-glycans and specifically demonstrate how sialic acids on N-glycans influence distinct functional outcomes in T cell activation. Our findings support cell-based glycoengineering as an effective strategy to improve DC-based immunotherapies.

## 1 Introduction

Dendritic cells (DCs) are major antigen-presenting cells essential for directing the response of the immune system toward tolerance or inflammation, through their unique ability to activate and prime naïve T cells. DCs achieve this by processing and presenting antigens through major histocompatibility complexes (MHC) type I and II (HLA-ABC and HLA-DR/DP/DQ, respectively) and by transmitting immunomodulatory signals via direct cell-to-cell interactions and cytokine secretion, thereby priming both CD4^+^ and CD8^+^ T cells (1–3). In addition to the functional changes that promote T cell activation during DC activation, the expression profile of N- and O-linked glycans, which are essential for glycoprotein folding, secretion, and stability, is also reshaped, suggesting a significant role for protein glycosylation in DC biology (4–6). As the most structurally diverse form of post-translational modification, glycosylation is becoming increasingly acknowledged for its essential roles in various processes within the human immune system (7–9).

Given the central role of DCs in coordinating immune responses, they are also key mediators of tumor-directed immunity. As central components of the tumor microenvironment (TME), DCs promote anti-tumor T cell responses, and their potent antigen presenting capacity has positioned them as a prominent target in cancer immunotherapy (1, 10, 11). Despite decades of research, DC-based cancer vaccines have yielded only modest clinical benefit. The first and only FDA-approved DC vaccine, sipuleucel-T (PROVENGE), is an autologous monocyte-derived DC (moDC) product that contains a mixture of various immune cells and has demonstrated inconsistent clinical performance, underscoring the challenges of advancing DC-based therapies to clinical success (12, 13, 14). Although DC vaccine strategies in clinical trials have induced antigen-specific T cell responses in some patients, the magnitude of the immune response, the functionality of activated T cells, and the establishment of long-lasting memory T cells still require significant improvement (11, 13, 15). One explanation for the suboptimal results is that current DC vaccines may lack sufficient immunogenicity to provoke clinically beneficial immune responses and overcome the immunosuppressive TME. Additionally, they may fail to induce the appropriate type of immunity; for instance, DCs might promote a TH_2_ response instead of a TH_1_ response (3, 12, 15–17). This highlights the need for new strategies to optimize DC function and immunogenicity. By enhancing the immunogenic properties of DCs, such as upregulating costimulatory molecules, boosting cytokine production, and improving antigen presentation capabilities, these DCs could potentially stimulate T cell responses more effectively, particularly stimulation of CD8^+^ T cells, which are crucial for eliminating cancer cells (13, 18). In addition to enhanced immunogenicity, an optimal DC vaccine should be easy to manufacture and ensure consistency in immunological activity, which are two challenges faced by autologous DC vaccines (19). Off-the-shelf allogeneic DC platforms can address these challenges while also reducing costs, production time, and overcoming the limited availability of autologous DC precursors. These advantages make allogeneic DC platforms particularly valuable, especially when combined with partial MHC class match (semi-allogeneic), as they can provide a reliable and reproducible therapeutic option that directly benefits patients (14, 20). Few off-the-shelf allogeneic DC-based platforms have been tested in clinical trials, but there are reports on DC cell lines that induce antigen-specific T cell responses (19–23). One example is the DC vaccine DCP-001, derived from the acute myeloid leukemia (AML) cell line DCOne, which has progressed to clinical trials for treatment of high grade serous ovarian cancer (NCT04739527) and AML (NCT03697707), currently in phase 1 and 2, respectively. The phase 2 study, involving 20 patients, reported findings suggesting that vaccinations promoted the expansion of DC populations, thereby boosting T cell activity against tumors (24). We recently developed and validated a human DC model system based on genetically glycoengineered MUTZ-3-derived DCs (25). The MUTZ-3 cell line originates from an AML patient, and MUTZ-3 progenitor cells (PCs) can be differentiated over a 7-day cytokine driven protocol into immature DCs (iDCs), that closely resemble moDCs in their surface receptors and gene expression profiles after cytokine-induced differentiation. MUTZ-3-derived iDCs are fully functional DCs capable of stimulating antigen-specific CD4^+^ and CD8^+^ T cells by processing and presenting protein antigens as peptides on MHC-I and MHC-II molecules, making this cell line a useful alternative model system for human moDCs (22, 26–32).

Previously, we validated the MUTZ-3 cell line as a suitable model for studying DC glycobiology and demonstrated that ST6GAL1-mediated α2,6-sialylation regulates the expression of various DC-related surface proteins, and selectively enhances CD4^+^ T cell activation, while having no effect on CD8^+^ T cell activation (25). These findings established the MUTZ-3 system a tractable isogenic model for dissecting the role of individual glycosyltransferases in DC biology and suggested that DC glycoengineering could be leveraged to optimize DC activation of T cells for future DC vaccine approaches. Moreover, combining *ST6GAL1* KO with enzymatic desialylation further enhanced CD8^+^ T cell activation, indicating the role of other sialic acid linkages in selective T cell activation (25). Collectively, previous studies have expanded our understanding of how sialylation modulates DC:T cell interactions and highlighted the potential of targeted glycan modification to fine-tune DC immunogenicity (4, 6, 25, 33–35).

**Figure 1:**
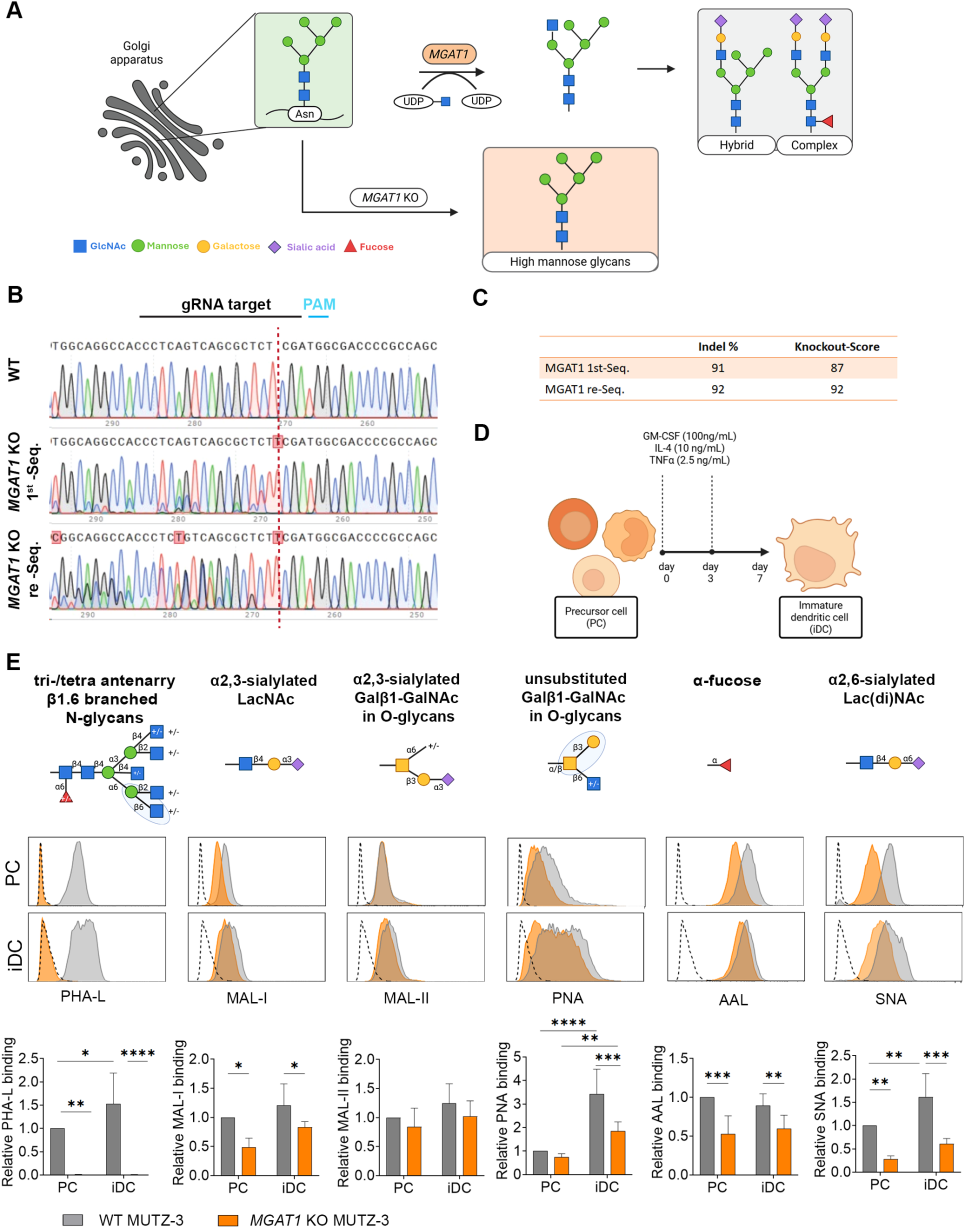
Successful and stable *MGAT1* KO in MUTZ-3 precursor cells (PCs) eliminates cell surface complex and hybrid N-glycans. **(A)** Simplified overview of MGAT1 function in N-glycan processing. MGAT1 initiates the conversion of high-mannose glycans to hybrid and complex N-glycans by adding a GlcNAc to the core structure. In the absence of MGAT1 (*MGAT1* KO), this step is blocked, resulting in accumulation of high-mannose glycans. Created in BioRender. Blomberg, A. (2025) https://BioRender.com/oheioep**(B)** Chromatograms displaying Sanger sequencing results for genotyping of MUTZ-3 WT and *MGAT1* KO pool. The gRNA target cleavage sites are marked by a vertical dashed red line. The initial sequencing (1st seq) and the subsequent sequencing (re-seq) were performed with 7 weeks of routine cell passaging in between. **(C)** Summary of Indel % and KO score for *MGAT1* KO PCs at initial sequencing (1st seq) and the subsequent sequencing (re-seq) performed with 7 weeks of routine cell passaging in between. **(D)** Illustration of MUTZ-3 PC differentiation protocol. Created in BioRender. Blomberg, A. (2025) https://BioRender.com/v5iyr9n. **(E)** WT and *MGAT1* KO PCs and iDCs were surface-labeled with various biotinylated lectins, followed by staining with AF488-streptavidin to assess the impact of *MGAT1* KO on cell surface glycan structures. The histograms display representative data from a single experiment, while the accompanying bar graphs show the mean ± SD of lectin intensities relative to WT PCs from seven independent experiments. Cells from different passages and differentiations were used across the seven experiments. The dotted histograms in each plot serve as the negative control, stained exclusively with AF488-streptavidin. Statistical analysis was conducted using two-way ANOVA on matched datasets, followed by Sidak’s multiple comparisons test to assess differences between WT cells and *MGAT1* KO cells. Statistical significance is denoted as *p < 0.05, **p < 0.01, ***p < 0.001, ****p < 0.0001. Glycan structures were created in BioRender. Blomberg, A. (2025) https://BioRender.com/h2ia4rl.

Building upon our previous work demonstrating the functional relevance of α2,6-linked sialic acids in DC biology, we next sought to investigate the broader consequences of depleting all N-linked sialic acids by targeting the central glycosyltransferase mannosyl (α-1,3-)-glycoprotein β-1,2-N-acetylglucosaminyltransferase (MGAT1*)*. MGAT1 initiates formation of hybrid and complex N-glycans via addition of a GlcNAc residue to the α3-linked mannose of the trimannosyl core of N-linked glycans. *MGAT1* KO therefore disrupts the synthesis of complex and hybrid N-glycans, resulting in an enrichment of high-mannose structures, specifically Man_5_GlcNAc_2_, and a complete loss of terminal sialylation on N-glycans, including both α2,3- and α2,6-linked sialic acids, but not on other glycans such as O-glycans and glycolipids (**Figure 1A**) (36). This makes *MGAT1* KO a powerful model to explore how the absence of broader glycan structures, including N-glycan sialylation and branching affects DC biology and T cell activation. This is to our knowledge, the first study to investigate how *MGAT1* KO, impacts human DC biology and function. By leveraging our isogenic DC model, we further elucidate how specific sialic acid linkages contribute to DC function, extending our previous findings on selective α2,6-sialylation (*ST6GAL1* KO) to a context where both α2,3 and α2,6 sialylation are absent selectively on N-glycans (25). In this study, we comprehensively characterize the phenotype and allogeneic T cell activation potential of MGAT1-deficient MUTZ-3 DCs.

## 2 Materials and methods

### 2.1 Cell culture and maintenance

The human acute myeloid leukemia-derived cell line MUTZ-3 (DSMZ, #ACC 295) and 5637 (DSMZ, #ACC 35) were cultured as previously described (25). Briefly, the MUTZ-3 cells were cultured in MEMα media (Gibco) supplemented with 20% FBS, 20% conditioned media from human cell line 5637 and 1% penicillin-streptomycin. Cells were passaged twice weekly at a density of 0.5×10^6^ cells/mL and maintained at 37°C in a 5% CO_2_ humidified incubator, with regular mycoplasma checks.

### 2.2 Differentiation of the MUTZ-3 DC

Differentiation of MUTZ-3 precursor cells (PCs) into immature dendritic cells (iDCs) was done as previously described (25, 31). Briefly, PCs were seeded into T75 flasks at a density of 1x10^5^ cells/mL in complete MEMα medium (MEMα+20%FBS+1% penicillin-streptomycin), supplemented with a cytokine cocktail consisting of 100 ng/mL GM-CSF (Peprotech), 10 ng/mL IL-4 (Peprotech), and 2.5 ng/mL TNFα (Peprotech) for 7 days. Fresh medium and cytokines were supplemented on day 3 or 4. Early immature dendritic cells (early iDCs) were generated following the same procedure, with the modification that cells were harvested on day 3 before cytokine replenishment.

### 2.3 CRISPR-Cas9 gene editing

*MGAT1* KO MUTZ-3 cells were generated by CRISPR-Cas9 gene editing as previously described (25). To summarize, validated guide RNA (gRNA) sequences were selected from a previous publication (37) and DNA oligos containing the gRNA sequence were synthesized by Macrogen Europe and ligated into U6GRNA plasmid (Addgene #68370). This plasmid, along with CAS9PBKS (Addgene #68371) containing Cas9-2A-EGFP, was transfected into MUTZ-3 cells. 48 hours post transfection cells were sorted based on GFP fluorescence using fluorescence-activated cell sorting (FACS). The KO was confirmed using Sanger Sequencing and ICE CRISPR Analysis Tool (Synthego), and selective lack of complex N-glycans on the KO cell surface was confirmed by lectin staining periodically as quality control of the cell lines in parallel to functional studies. To mitigate any unintended phenotypic or genetic alterations introduced during the gene editing process, WT cells were treated under identical conditions as *MGAT1* KO cells, including freeze/thaw cycles and passage numbers.

### 2.4 Glycan profiling by lectin staining

The selective lack of complex N-glycans on the *MGAT1* KO cell surface was confirmed by lectin staining as previously described (25). Briefly, cells were stained with LIVE/DEAD™ Fixable Yellow Dead Cell Stain to assess viability, before incubation with a selection of biotinylated lectins from Vector Laboratories: Aleuria aurantia lectin (AAL), Maackia amurensis lectin I and II (MAL-I and MAL-II), Sambucus nigra agglutinin (SNA), Phaseolus vulgaris lectin L (PHA-L) and Peanut agglutinin (PNA). Detection was achieved using AF488-conjugated streptavidin (Invitrogen), and both lectins and streptavidin were incubated with the cells on ice for 20 min. Detailed information on the working concentrations and catalogue numbers of the lectins is provided in **Supplementary Table S1**. The cells were acquired on MACSQuant^®^ Analyzer 16 (Miltenyi Biotec) and data was analyzed in FlowJo v10.8.1.

### 2.5 Microfludic high-throughput qPCR

Cells were lysed, and RNA was extracted as previously described (25). The purity, concentration, and quality of the extracted RNA were assessed using a NanoDrop™ One/OneC Microvolume UV–Vis Spectrophotometer (Thermo Fisher) and an Agilent 2100 Bioanalyzer (Agilent Technologies), respectively. Complementary DNA (cDNA) synthesis and the pre-amplification step were carried out analogously to the previously described method (25), with the following exceptions: cDNA was synthesized using 300 ng of total RNA, and amplification was performed by incubation at 95°C for 10 minutes, followed by 20 cycles of 95°C for 10 seconds and 60°C for 4 minutes. The specific primes are listed in **Supplementary Table S2**.

Microfluidic high-throughput qPCR was performed using a 96.96 Dynamic Array Integrated Fluidic Circuit (IFC) chip (Standard Biotools) on the BioMark real-time PCR instrument (Standard Biotools), as previously described (25). Data were normalized using *GAPDH* and *RPLP0* after evaluation of reference genes with NormFinder (38) and geNorm (39). Technical replicates were averaged, and relative quantities were calculated based on the lowest expressed sample for each assay. The graphs depicted are based on log-transformed data, generated using RStudio.

### 2.6 Isolation of primary cells and coculture of MUTZ-3 iDCs and T cells

Buffy coats were obtained from nine anonymized healthy donors with informed written consent, following local ethics committee guidelines (Region Hovedstaden, Denmark). Peripheral blood mononuclear cells (PBMCs) were isolated using a two-step density gradient centrifugation (200xg and 460xg) with Histopaque-1077 (Sigma Aldrich). PBMCs were collected from the interphase, washed multiple times with Dulbecco’s PBS (DPBS), and depleted for any leftover red blood cells using red blood cell lysis buffer (eBioscience). T cells were subsequently isolated from the PBMC fraction using Dynabeads Untouched Human T cell kit according to the manufacturer’s protocol (Invitrogen). For proliferation analysis, purified T cells were incubated with 5 µM CellTrace™ Violet Cell Proliferation Kit (Invitrogen) in PBS with 5% FBS for 5 minutes, followed by two washes with PBS and 5% FBS. T cells were cocultured with MUTZ-3-derived iDCs or early iDCs in U-bottom 96-well plates at a T cell to DC ratio of 10:1 in complete RPMI 1640 (Sigma Aldrich) with a seeding density of 1x10^6^ T cells/mL. In indicated experiments, an in-house–produced non-glycosylated IgG1 anti–PD-L1 (aPD-L1) antibody was added at a final concentration of 1 µg/mL (see below). After 5 days of coculture at 37°C in a humidified incubator with 5% CO2, T cell activation was assessed by flow cytometry (detailed in the Materials and Methods section on phenotyping DCs and T cells). Supernatants were collected on day 5 of coculture and stored at -80°C for further analysis.

### 2.7 Cytokine profiling of supernatants

Supernatants from iDC and T cell cocultures were examined for cytokine content using V-Plex Proinflammatory Panel 1 (human) and V-Plex Cytokine Panel 1 (human) MesoScale Discovery (MSD) multiplex assays. Sample preparation followed the manufacturer’s guidelines, and the analysis was conducted using MSD Sector Imager. Data from these assays were processed and analyzed with Discovery Workbench software v4.

### 2.8 Phenotyping of DCs and T cells

Cells were stained in a 96-well U-bottom plate at 0.5-1x10^5^ cells/well in FACS buffer (PBS + 2% FBS + 2mM Ethylenediaminetetraacetic acid (EDTA)) and were washed in between incubations. Cells were first incubated for 10 min with LIVE/DEAD™ Fixable Yellow Dead Cell Stain (Invitrogen) followed by 10 min incubating with FcR Blocking Reagent (Miltenyi) and finally 10 min incubation with fluorescently labeled detection antibodies. All incubation steps were performed cold in the dark. The antibodies are listed in **Supplementary Table S3**. After staining, cells were fixed with Fixation buffer (eBioscience) for later analysis. For surface staining, gating was based on the relevant fluorescence minus one (FMO) control on pooled samples. All samples were analyzed on MACSQuant^®^ Analyzer 16 (Miltenyi Biotec), and data further compensated and analyzed in FlowJo version 10.8.1.

### 2.9 aPD-L1 antibody

The antibody directed against PD-L1 (aPD-L1) was generated as previously described (40, 41). Briefly, the variable heavy (VH) and light (VL) domains of atezolizumab were cloned into the constant domains of a human IgG1 using pcDNA3.1-based expression vectors. To generate an Fc-silent variant, a point mutation (N297A) was introduced in the heavy-chain CH2 domain using Q5 site-directed mutagenesis kit (New England Biolabs) to eliminate N-linked glycosylation and Fc-mediated effector functions. Heavy- and light-chain plasmids were co-transfected into CHO-S cells to establish a stable expression pool, and antibodies were purified using Protein A affinity chromatography. Endotoxin levels were <0.2 EU/mg, and antibody purity was verified by SDS–PAGE. Binding affinity (K_D_) of aPD-L1 to human PD-L1 (ACROBiosystems, #PD1-H52H3) were measured by biolayer interferometry (BLI) on an Octet RED96e system (FortéBio). Antibodies (1.5 µg/mL in PBS, pH 7.4, 0.02% Tween-20, 0.1% BSA) were immobilized on AHC biosensors (Sartorius), associated with PD-L1 (0–5 nM) for 600 s, and allowed to dissociate for 2400 s. Data were analyzed using ForteBio Data Analysis Software 12.0, aligning to the association step and applying a Savitzky–Golay filter to globally fit sensorgrams to a 1:1 binding model (n = 3). Atezolizumab (Tecentriq^®^) was included as a control.

## 3 Results

### 3.1 Successful and stable *MGAT1* KO in MUTZ-3 precursor cells eliminates cell surface complex and hybrid N-glycans

The MUTZ-3 *MGAT1* KO cell line was generated utilizing the CRISPR-Cas9 mediated genetic engineering strategy, previously published by our group (25). The *MGAT1* KO cell pool was analyzed for KO percentage using Sanger sequencing 3- and 10 weeks post FACS (**Figure 1B**). The *MGAT1* KO was confirmed with an initial KO score of 87%, followed by a second score of 92% after a 7-week culture period (**Figure 1C**). The heterogeneous bulk *MGAT1* KO cell pool and WT PCs were utilized for subsequent experiments.

To evaluate the functional impact of *MGAT1* KO on the cell surface glycoprofile of MUTZ-3 PCs and iDCs, WT and *MGAT1* KO PCs were differentiated for 7 days with GM-CSF, IL-4, and TNFα (**Figure 1D**), and both the PCs and iDCs were stained with a series of lectins and analyzed by flow cytometry (**Figure 1E**). The glycan specificities of the lectins used are depicted above each set of histograms in **Figure 1D** (42). Monosaccharide symbols follow the SNFG (Symbol Nomenclature for Glycans) system (43). The successful functional *MGAT1* KO was confirmed by PHA-L staining, which recognizes tri– and tetraantennary complex N-glycans and demonstrated complete loss of binding. This was further confirmed by the reduced binding of SNA, recognizing α2,6-sialylated Lac(di)NAc, MAL-I, recognizing α2,3-sialylated Lac(di)NAc, and AAL, recognizing α-fucose. These reductions reflect the depletion of complex N-glycans, leading to diminished terminal sialylation and fucosylation. Because SNA, MAL-I, and AAL also bind O-glycans and are not exclusive to N-glycans, a complete loss of binding was neither observed nor anticipated (42). No difference in O-glycan binding MAL-II was observed between *MGAT1* KO and WT cells on either PC or iDC, whereas a significant reduction in PNA binding was observed in *MGAT1* KO iDCs compared to WT iDCs.

### 3.2 *MGAT1* KO induces enhanced surface density of HLA-ABC, HLA-DR, and CD40 upon differentiation to iDCs alongside increased mRNA levels of pro-inflammatory regulators

**Figure 2:**
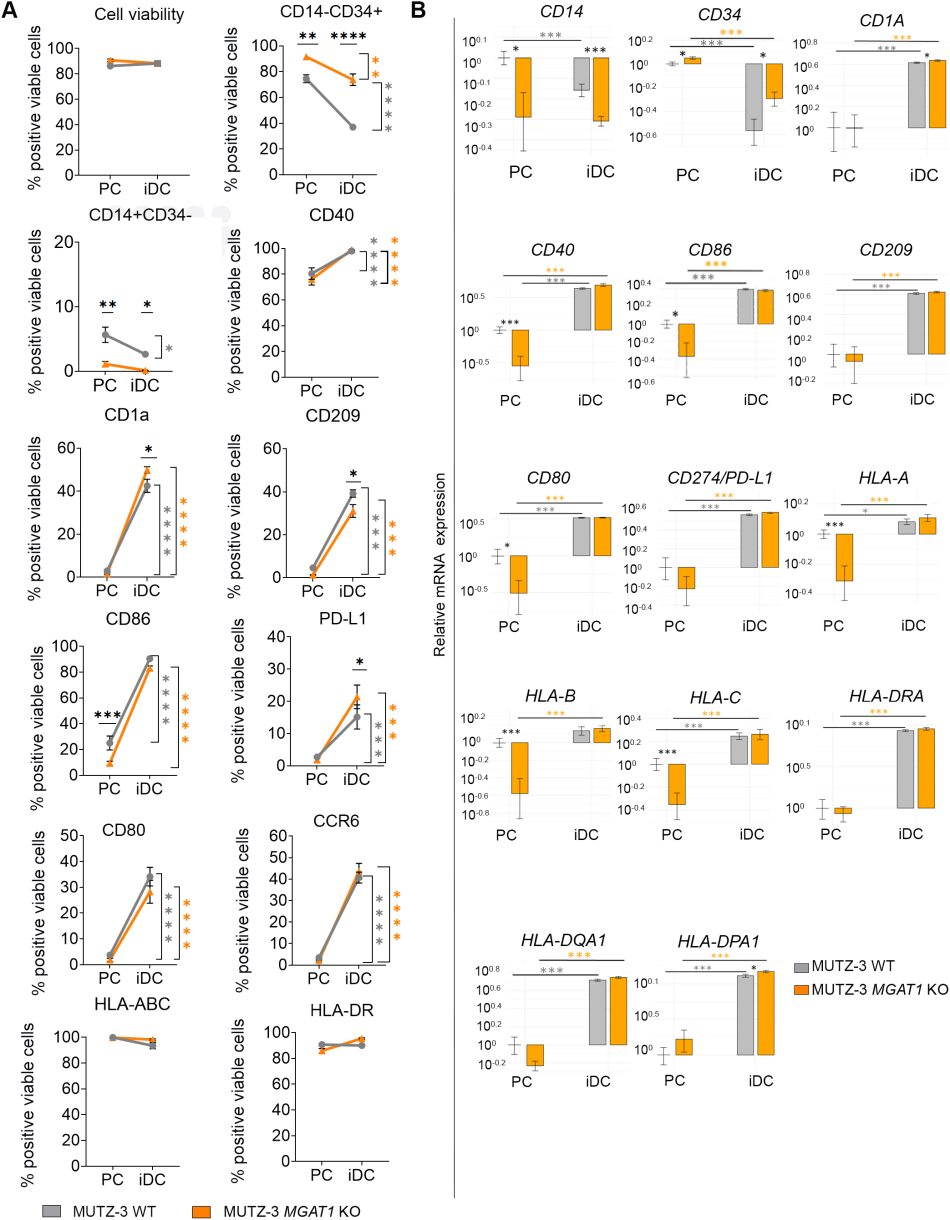
*MGAT1* KO PCs effectively differentiate into *MGAT1* KO iDCs by regulating expression of various DC related receptors both on protein and mRNA level. **(A)** MUTZ-3 WT PCs and MUTZ-3 *MGAT1* KO PCs were differentiated into iDCs, with phenotypic characterization at each stage performed by flow cytometry for a panel of DC markers. Data is presented as percentage of positive viable cells, illustrating the development of indicated surface markers from PC to iDC stage. Statistical analysis was conducted using a two-way ANOVA on paired datasets (n=3-6), followed by Sidak’s multiple comparisons test to evaluate differences between PCs and iDCs and the difference between WT and *MGAT1* KO. Statistical differences for *MGAT1* KO PCs versus iDCs are indicated by orange asterisk, for WT by grey asterisk, and for comparisons between WT and *MGAT1* KO at both PC and iDC stages by black asterisk. Significance is denoted as *p < 0.05, **p < 0.01, ***p < 0.001, ****p < 0.0001. **(B)** Bar graphs depict the transcriptional regulation of the same DC markers analyzed by flow cytometry (n=6–7). Bars represent mean mRNA levels ± SEM. Asterisks denote statistically significant differences between samples, determined by Student’s t-test (*P < 0.05, **P < 0.01, ***P < 0.001).

MUTZ-3 PCs are known to comprise three distinct subpopulations based on CD34 and CD14 surface expression (25, 31). The CD34^+^CD14^−^ subpopulation represents a stem cell-like, proliferative fraction that differentiates through a CD34^−^CD14^−^ intermediate stage to form the CD34^−^CD14^+^ subpopulation, which acts as an immediate precursor to MUTZ-3 derived DCs (31). When comparing WT and *MGAT1* KO cells, *MGAT1* PCs exhibited fewer CD34^-^CD14^+^ cells and more CD34^+^CD14^-^ PCs (**Figure 2A**). However, as a response to differentiation both WT and *MGAT1* iDCs significantly downregulated CD34^+^ expression as expected. DC cell surface markers CD1a, CD80, CD86, CD209, CCR6 and PD-L1 were all detected at very low levels in WT and *MGAT1* KO PCs by flow cytometry. Upon exposure to the differentiation cocktail, all markers were significantly upregulated to comparable levels in both WT and *MGAT1* KO cell lines. Notably, CD1a and PD-L1 expression was significantly higher on *MGAT1* KO iDCs compared to WT iDCs, while CD209 expression was significantly lower on *MGAT1* KO iDCs compared to WT iDCs (**Figure 2A**). CD40, which was already highly expressed at PC level, also increased further after differentiation. Again, a similar response between WT and *MGAT1* KO cells was observed regarding regulation of this DC cell surface receptor (**Figure 2A**). Gene expression-levels of CD14, CD34, CD1A, CD40, CD86, CD209, CD274/PD-L1, HLA-A/B/C, and HLA-DRA/DPA/DQA were consistent with expression patterns observed by flow cytometry (**Figures 2A, B**). Notably, the differences in CD14, CD34, CD1A, and CD86 surface expression on *MGAT1* KO PCs and iDCs compared to WT were also reflected at the transcriptional level, confirming that the observed differences are transcriptionally regulated rather than due to glycosylation-dependent differences in detection antibody binding.

**Figure 3:**
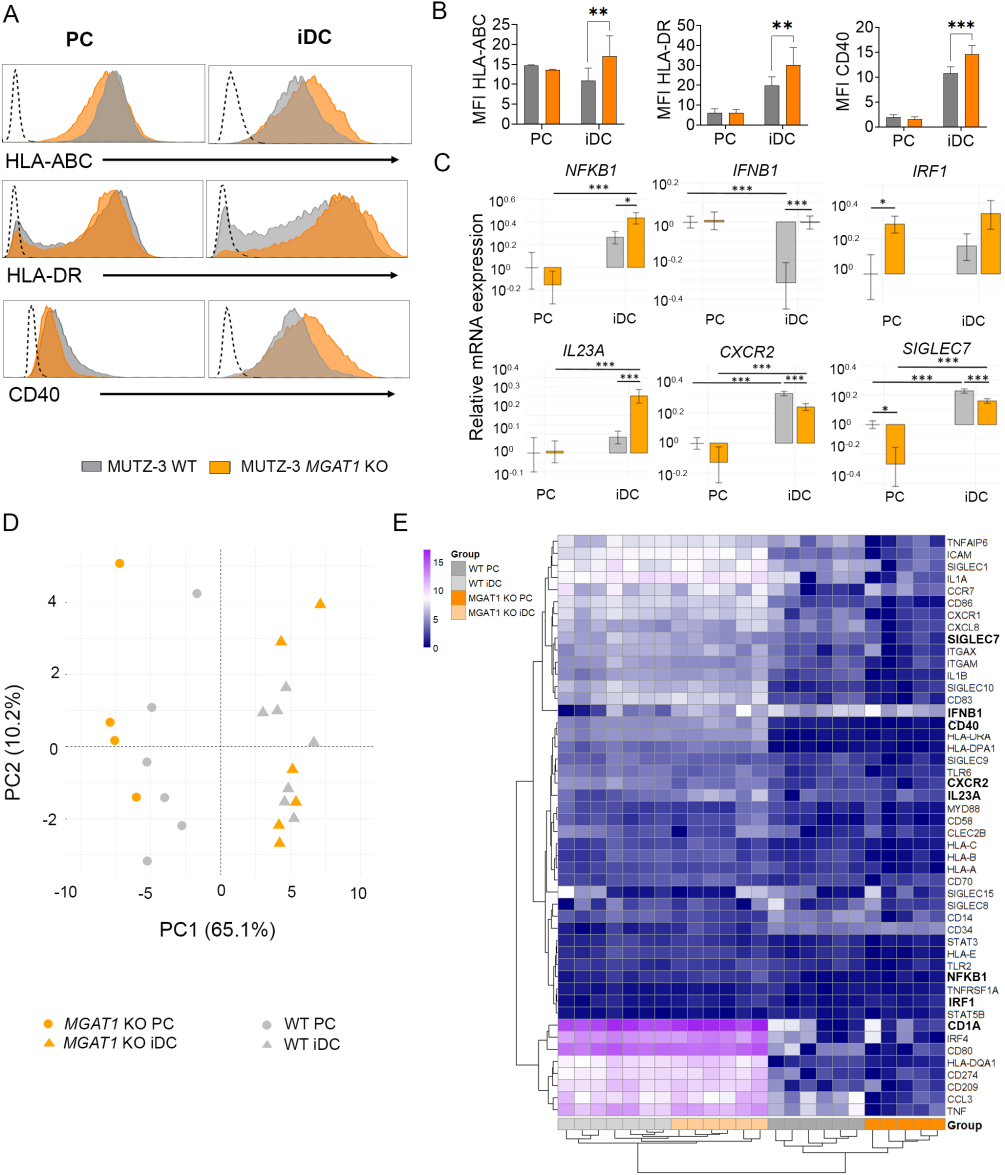
*MGAT1* KO iDCs display increased MFI of HLA-ABC, HLA-DR and CD40 molecules and elevated mRNA levels of pro-inflammatory regulators. **(A)** Representative histograms showing mean fluorescence intensities (MFI) for HLA-ABC, HLA-DR, and CD40 in PCs and iDCs for WT cells and *MGAT1* KO cells. The dotted line represents FMO control. **(B)** Bar graphs showing MFI for DC receptors that are expressed by ~100% if the DCs. Statistical analysis was conducted using two-way ANOVA on paired datasets, followed by Sidak’s multiple comparisons test to assess differences between WT cells and *MGAT1* KO cells on PCs and iDC state. Bar graphs represent 5–7 independent experiments. **(C)** Bar graphs depict transcriptional regulation of select genes, highlighting statistically significant differences between WT and MGAT1 KO at the iDC stage (n=6–7), with bars showing mean mRNA levels ± SEM. Statistical significance was assessed by Student’s t-test (*P < 0.05, **P < 0.01, ***P < 0.001). **(D)** Principal component analysis (PCA) of WT and *MGAT1* KO cells at PC and iDC stage based on data from transcript analysis. The PCA analysis is based on 47 genes related to DC phenotype and function. The specific genes are indicated in **Figure 3D** and Supporting Information **Supplementary Table S2**. **(E)** Heatmap of gene expression for 47 selected genes related to DC function and phenotype. Genes in bold represent the genes with differential expression between *MGAT1* KO iDCs and WT iDCs.

The differentiation into iDCs did not alter the percentage of cells positive for HLA-ABC and HLA-DR in either WT or *MGAT1* KO cells, with nearly 100% of both PCs and iDCs expressing HLA-ABC, HLA-DR, and CD40 (**Figure 2A**). However, significant differences in mean fluorescence intensity (MFI), which reflects the cell surface density of the analyzed receptors, were observed between WT and *MGAT1* KO iDCs; notably, *MGAT1* KO iDCs displayed significantly higher MFI levels for HLA-ABC, HLA-DR, and CD40 compared to WT iDCs (**Figures 3A, B**).

In addition to assessing phenotypical differences between WT and *MGAT1* KO cells, we conducted qPCR analysis on a broader panel of genes associated with DC activation and maturation (**Figure 3E**, **Supplementary Table S2**). Principal Component Analysis (PCA) of gene expression profiles revealed that WT and *MGAT1* KO groups were not separated at either the PC or iDC stage, indicating similar overall transcriptional patterns between WT and *MGAT1* KO within each stage (**Figure 3D**). This is consistent with flow cytometry data where no large phenotypic differences were observed between the groups at either stage (**Figure 2A**). However, the PCA revealed a clear separation between the PC and iDC stages, regardless of WT or MGAT1 KO status, reflecting distinct transcriptional differences associated with these developmental stages (**Figure 3D**). Closer examination at the expression of individual genes at the iDC stage revealed strongly upregulated mRNA levels of *NFKB1* and *IL23A*, moderate upregulation of *IFNB1*, and upregulation of *IRF1* (though not statistically significant) in *MGAT1* KO iDCs compared to WT iDCs. In contrast, strong downregulation of *SIGLEC7* and *CXCR2* expressions were also detected in *MGAT1* KO iDCs (**Figure 3C**).

### 3.3 *MGAT1* KO iDCs strongly enhance activation of CD8^+^ T cells and cytokine production after coculture

**Figure 4:**
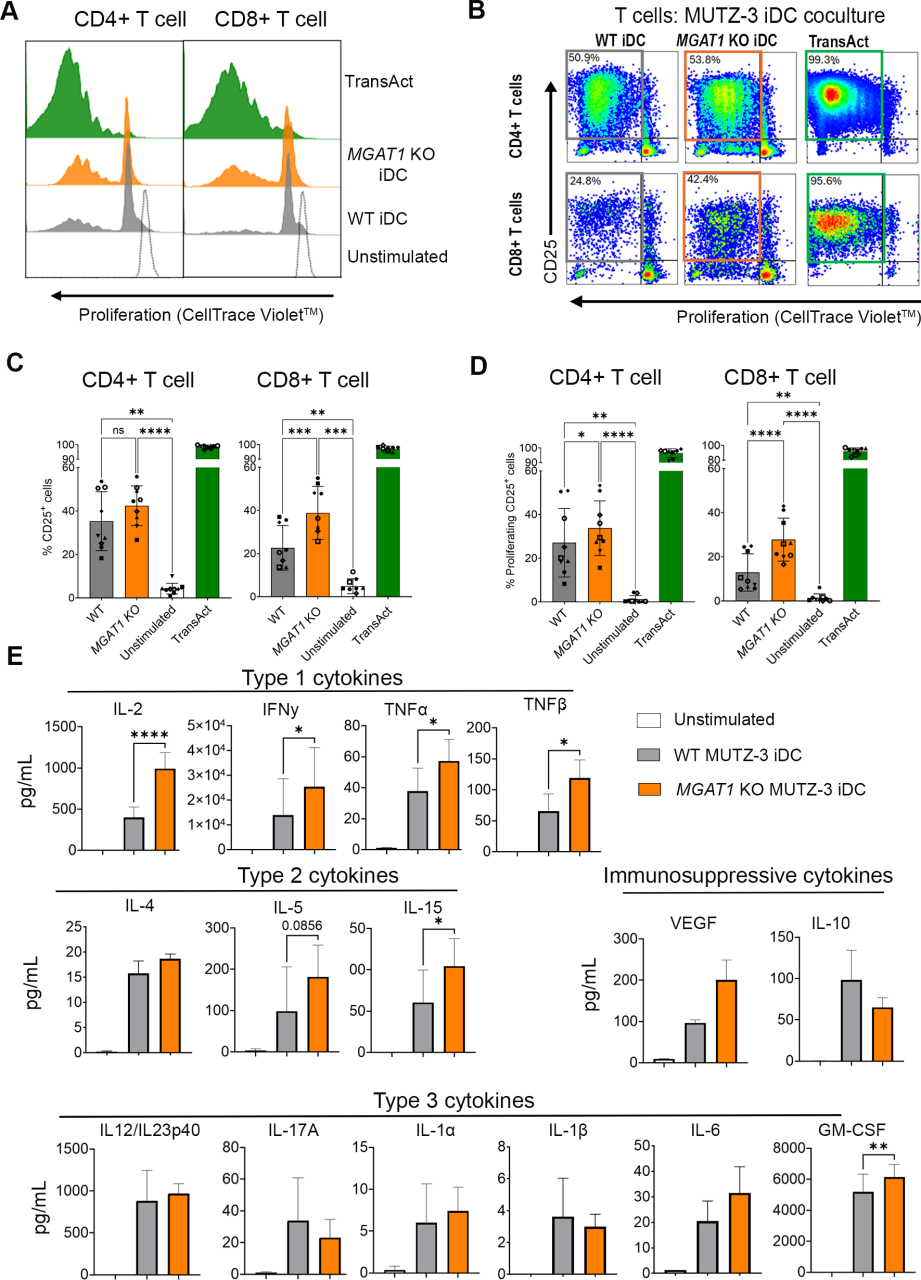
*MGAT1* KO in iDCs enhance CD25 expression and proliferation of allogenic CD8^+^ T cells and increase secretion of Type 1 cytokines. WT and *MGAT1* KO iDCs were cocultured with allogenic primary T cells for 5 days, after which the activation of the T cells was measured by evaluation of their proliferation via Cell Trace Violet (CTV) staining and cell surface expression of CD25 on CD4^+^ and CD8^+^ T cell subpopulations. **(A)** Histograms displaying proliferation of CD4^+^ and CD8^+^ T cells after coculture. Green histograms indicate T cells activated with TransAct™ for full activation. Dotted histograms displaying unstimulated T cells. **(B)** Representative dot plots of CD8^+^ T cells from one donor showing the difference in the CD25^+^ proliferating subpopulation between cells cocultured with WT and *MGAT1* KO iDCs. **(C, D)**. **(C, D)** Bar plots summarize the overall percentage of CD25^+^ cells and the percentage of proliferating CD25^+^ cells within each T cell subpopulation from nine different donors. Statistical analysis was performed on matched donor datasets using repeated-measures (RM) one-way ANOVA with the Geisser–Greenhouse correction, followed by Tukey’s multiple comparisons test. Significance is denoted as *p* < 0.05, *p* < 0.01, *p* < 0.001, and *p* < 0.0001. **(E)** Cytokine levels in supernatants after 5 days of coculture between T cells and WT or *MGAT1* iDC. **(-)** shows cytokine levels of unstimulated T cells. Statistical analysis of differences between WT and *MGAT1* KO was performed using a paired two-tailed ratio-t test. (*p < 0.05, **p < 0.01, and ***p < 0.001). The functional categorization of cytokines and chemokines are based on (44, 45). ns: not significant.

Phenotyping of WT and *MGAT1* KO PCs and iDCs revealed similar regulatory patterns across various DC markers upon differentiation (**Figure 2A**). However, HLA-ABC, HLA-DR, and CD40 showed significantly enhanced surface density on *MGAT1* KO iDCs compared to WT iDCs (**Figures 3A, B**). Additionally, elevated expression levels of specific pro-inflammatory genes were also observed in *MGAT1* KO iDCs including *NFKB1 and IFNB1* (**Figure 3C**). Given the importance of these receptors and genes in DC maturation and T cell priming, we next examined whether coculturing primary T cells with WT or *MGAT1* KO iDCs would result in differences in T cell activation. After 5 days of coculture between iDCs and primary T cells, CD4^+^ and CD8^+^ T cell activation was assessed by examining CD25 surface expression and proliferative capacity (**Figures 4A–D**). No differences in CD25 expression were initially observed on CD4^+^ T cells after coculture with WT or *MGAT1* KO iDCs (**Figures 4A, C, D**. However, closer analysis revealed a modest but statistically significant increase in the proportion of proliferating CD25^+^ CD4^+^ T cells in *MGAT1* KO iDC cocultures compared with WT (**Figure 4D**). The magnitude of this difference varied between donors, with some showing a clear enhancement in response to *MGAT1* KO iDCs and others displaying minimal change. In contrast, the CD8^+^ T cell subset showed a strong, robust, and reproducible increase in both CD25 expression and the frequency of proliferating CD25^+^ cells following coculture with *MGAT1* KO iDCs compared with WT, representing a marked enhancement of CD8^+^ T cell activation (**Figures 4A–D**). The supernatants from the cocultures (day 5) were investigated by Mesoscale to determine the resulting cytokine profile (**Figure 4E**). The increased CD8^+^ T cell activation was supported by the cytokine profile where the *MGAT1* KO coculture had significantly higher levels of IL-2, IFNγ, TNFα and TNFβ. Additionally a significant increase in GM-CSF was observed in *MGAT1* KO iDC cocultures together with an increased level of IL-13. No significant differences were observed in the other tested cytokines.

### 3.4 *MGAT1* KO iDCs maintain enhanced CD8^+^ T cell activation under PD-L1 blockade

**Figure 5:**
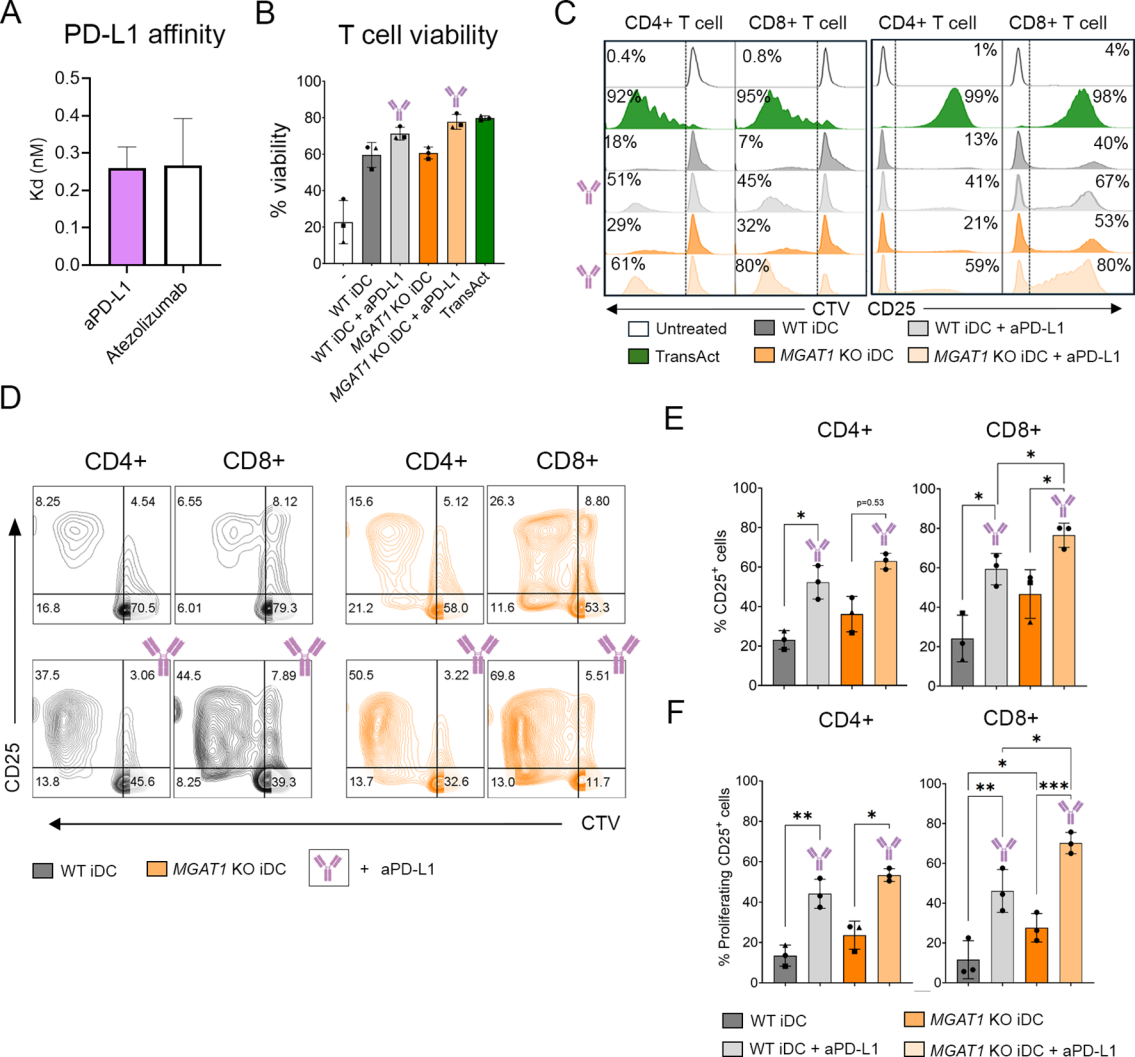
PD-L1 blockade enhances overall T cell activation while maintaining stronger CD8^+^ T cell responses induced by *MGAT1* KO iDCs. **(A)** Bar graphs illustrating the mean PD-L1 K_D_ values (nM) of in-house produced aPD-L1 and commercial atezolizumab **(B)** T cell viability after 5 days of coculture between iDCs and primary T cells in the presence or absence of aPD-L1 is shown as the percentage of viable cells for each condition. **(C, D)** T cell proliferation (CellTrace Violet) and CD25 expression of CD4^+^ and CD8^+^ T cells after 5 days of coculture with WT or *MGAT1* KO iDCs in the presence or absence of aPD-L1 are shown as representative histograms **(C)** and contour plots **(D)** from one donor, with numbers indicating the percentage of cells within the respective gates. **(E, F)** Bar plots summarize the overall percentage of CD25^+^ cells **(E)** and proliferating CD25^+^ cells **(F)** within each T cell subpopulation after 5 days of coculture with WT or *MGAT1* KO iDCs in the presence or absence of aPD-L1. Statistical analysis was performed on matched donor datasets using repeated-measures (RM) one-way ANOVA with the Geisser–Greenhouse correction, followed by Tukey’s multiple comparisons test (n = 3) (*p < 0.05, **p < 0.01, and ***p < 0.001).

To increase the T cell activation in the cocultures, we applied PD-L1 blockade using an anti–PD-L1 antibody (aPD-L1). Both WT and *MGAT1* KO iDCs expressed PD-L1 (**Figure 2A**), providing the rationale for applying PD-L1 blockade to further enhance T cell activation. The aPD-L1 is based on the variable domains of atezolizumab fused to human IgG1 Fc mutated at position 297 in CH2 domain to lack the glycosylation which leads to abrogation of Fc mediated immune effector functions, including lack FcγR (CD16) binding, but retaining full binding affinity and PD1 axis immune blockade. As shown in **Figure 5A**, our aPD-L1 exhibited binding kinetics comparable to atezolizumab, with similar K_D_ values determined by BLI. After 5 days of coculture between iDCs and T cells in the presence of aPD-L1, a minor but statistically significant increase in T cell viability was observed in *MGAT1* KO iDC cocultures compared to WT cocultures, although overall viability remained high across all conditions (**Figure 5B**). As expected, aPD-L1 treatment markedly enhanced T cell activation, increasing CD25 expression and proliferation in both CD4^+^ and CD8^+^ populations across WT and *MGAT1* KO cocultures (**Figures 5C–F**). Despite this increase in overall activation, and a tendency for slightly improved CD25 expression and proliferation, no significant differences were observed between WT and *MGAT1* KO iDC cocultures within the CD4^+^ T cell subset (**Figures 5E, F**). In contrast, CD8^+^ T cells exhibited consistently stronger responses in *MGAT1* KO iDC cocultures, with significantly higher frequencies of CD25^+^ and proliferating CD25^+^ cells compared to WT, both with and without aPD-L1 treatment (**Figures 5E, F**). These data show that PD-L1 blockade successfully enhances global T cell activation in our MUTZ-3 DC model, and the stronger CD8^+^ T cell response induced by *MGAT1* KO iDCs remains evident under PD-L1–inhibited conditions.

### 3.5 *MGAT1* KO accelerates differentiation of MUTZ-3 PCs into functionally competent DCs

**Figure 6:**
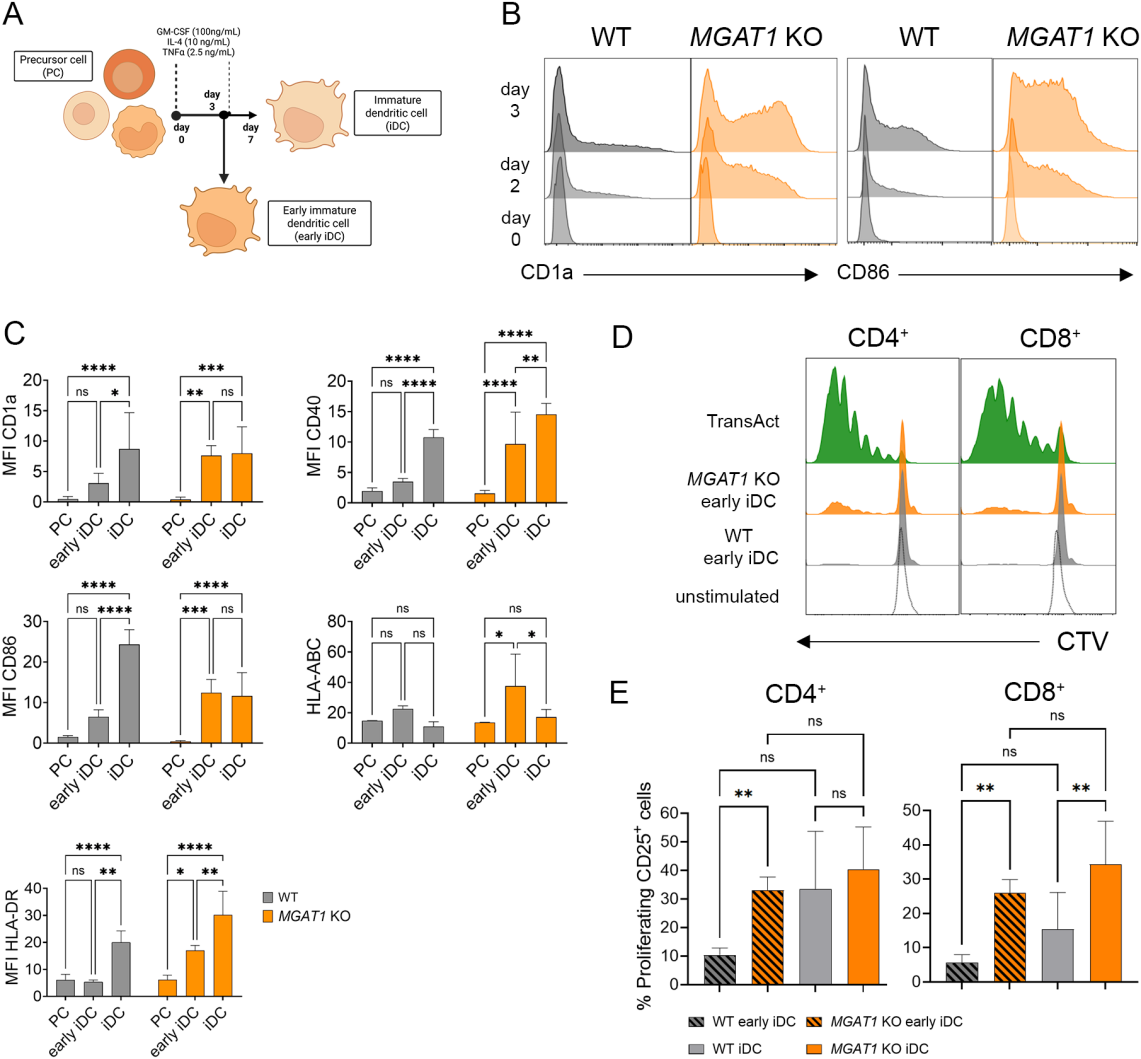
*MGAT1* KO accelerates MUTZ-3 differentiation toward functionally competent DC. **(A)** Schematic overview of MUTZ-3 differentiation into immature dendritic cells (iDCs), illustrating the generation of iDCs harvested on day 3 (early iDC) and iDCs harvested on day 7 (iDC). Created in BioRender. Blomberg, A. (2025) https://BioRender.com/cbe7i13**(B)** Expression of early DC markers CD1a and CD86 in WT and *MGAT1* KO cells after 2 days of cytokine-induced differentiation, presented as the percentage of positive viable cells. **(C)** Mean fluorescence intensity (MFI) of DC-associated surface markers (CD1a, CD40, CD86, HLA-ABC, and HLA-DR) measured in WT and *MGAT1* KO cells at the precursor (PC), early iDC (day 3), and iDC (day 7) stages. **(D, E)** Activation and proliferation of CD4^+^ and CD8^+^ T cells after 5 days of coculture with either early iDCs or iDCs derived from WT or *MGAT1* KO MUTZ-3 cells. Data are presented as **(D)** representative histograms showing proliferation (CellTrace Violet dilution) and CD25 expression and **(E)** bar plots summarizing the percentage of CD25^+^ and proliferating CD25^+^ cells within each T cell subpopulation. Statistical analysis was performed on matched donor datasets using repeated-measures (RM) one-way ANOVA with the Geisser–Greenhouse correction, followed by Tukey’s multiple comparisons test (n = 3). (*p < 0.05, **p < 0.01, ***p < 0.001) and ****p<0.0001).

Because *MGAT1* KO iDCs elicited stronger T cell activation (**Figures 5C–F**) despite few phenotypic differences after 7 days of differentiation (**Figures 2A**, **3A**), we next asked whether *MGAT1* deficiency also exerts its effects at earlier stages of DC differentiation. To explore this, we harvested iDCs after 3 days of differentiation and characterized these early immature DCs (early iDCs) (**Figure 6A**). Phenotypic analysis revealed that *MGAT1* KO cells responded rapidly to the cytokine cocktail and upregulated DC-associated surface markers, including CD1a and CD86, already by day 2 which was not the case with WT cells (**Figure 6B**). By day 3, *MGAT1* KO early iDCs displayed a clear phenotypic shift, with significantly increased expression of CD1a, CD40, CD86, HLA-ABC, and HLA-DR compared with PCs, whereas WT cells showed only minor, non-significant upregulation at this early stage (**Figure 6C**). In contrast, WT cells required the full 7-day differentiation period to reach a significant upregulation of DC markers compared with their PC level, indicating that *MGAT1* deficiency accelerates the transition of PCs toward a DC phenotype. To determine whether these phenotypic differences translated into functional competence, early iDCs were cocultured with primary T cells for 5 days. *MGAT1* KO early iDCs induced pronounced proliferation of both CD4^+^ and CD8^+^ T cells, whereas WT early iDCs elicited only minimal proliferation, indicating a markedly much lower capacity to stimulate T cell activation (**Figures 6D, E**). This low responsiveness in WT cocultures correlates with their limited upregulation of DC surface markers at the early differentiation stage. In contrast, *MGAT1* KO early iDCs promoted robust T cell activation, with a significantly higher proportion of proliferating CD25^+^ T cells compared with WT.

## 4 Discussion

In this study, we generated a stable and functional *MGAT1* KO MUTZ-3 cell line to examine how oligomannose N-glycosylation impacts human DC biology and function. We previously demonstrated that a functional KO of *ST6GAL1* in the MUTZ-3 cell line significantly altered the phenotype of MUTZ-3-derived DCs and significantly increased CD4^+^ T cell activation, but not CD8^+^ T cell activation (25). To confirm that these effects were linked specifically to the *ST6GAL1* KO and not an artifact of the KO procedure, we created a *FUT8* KO MUTZ-3 DC pool, which exhibited T cell activation levels comparable to WT (25). Furthermore, in this study the combination of the *ST6GAL1* KO in MUTZ-3 with additional enzymatic desialylation lead to an additional boost of activation of CD8^+^ T cells yielding both enhanced activation of CD4^+^ and CD8^+^ T cell activation indicating the role of other sialic acid linkages in selective T cell activation. These previous findings indicate that glycoengineering of DCs may represent a promising approach to modulate DC phenotype and function, thereby enhancing their therapeutic potential.

The *MGAT1* KO MUTZ-3 PCs generated in this study showed abolished PHA-L binding, proving the absence of all complex N-glycans on the cell surface (42). Despite the significantly distinct glycoprofiles between WT and *MGAT1* KO PCs (**Figure 1E**), both cell lines successfully differentiated into iDCs, following a 7 day differentiation protocol (**Figure 1D**), characterized by the downregulation of CD14 and CD34 and the upregulation of CD1a, CD40, CD80, CD86, CCR6, CD209, and PD-L1 at both protein and transcriptional level (**Figures 2A, B**). At the iDC stage, nearly 100% of both WT and *MGAT1* KO iDCs were positive for HLA-ABC, HLA-DR, and CD40, however, the cell surface density of these molecules was significantly higher in *MGAT1* KO compared to WT iDCs (**Figures 3A, B**). All HLA family members are known to carry N-linked glycans (46, 47) and this glycosylation is essential for proper HLA protein folding and surface expression (48). A study by Silva et al., (49) examined the effects of enzymatic desialylation on both lymphoblastoid cells and moDCs, demonstrating that desialylation resulted in increased surface expression of MHC-I complexes and a twofold extension of the half-life of MHC-I molecules at the cell surface in both cell types. The functional impact of this desialylation of moDCs was further evaluated in a mixed leukocyte reaction (MLR) with autologous CD8^+^ T cells, where desialylation enhanced IFNγ production by the T cells. Our findings complement this work by showing that *MGAT1* KO iDCs have enhanced surface MHC-I expression (**Figures 3A, B**) and allogenic T cells cocultured with *MGAT1* KO iDCs exhibit increased IFNγ production. In the *MGAT1* KO DCs, all N-glycan sialic acids are depleted, as they do not occur on the remaining high mannose structures, while sialic acids on O-glycans and glycolipids are not directly affected.

The coculture between *MGAT1* KO iDCs and allogenic T cells also led to a significantly increased activation of CD8^+^ T cells, marked by upregulated CD25 expression, enhanced proliferation and elevated secretion of proinflammatory cytokines including TNFα, TNFβ, and IL-2, in addition to IFNγ (**Figure 4E**). These cytokines are all known to be secreted by CD8^+^ T cells, thereby reflecting the increased CD8^+^ T cell activation (44, 50). The increased cell surface density of HLA-ABC could partially explain this. While an increased HLA-DR MFI was also observed, the effect on CD4^+^ T cells was less pronounced and appeared more variable between donors. A modest but statistically significant increase in proliferating CD25^+^ CD4^+^ T cells was detected in *MGAT1* KO cocultures compared with WT, whereas total CD25^+^ frequencies did not differ significantly. This variability suggests that CD4^+^ T cell activation is more donor dependent, with some individuals showing stronger responses than others, in contrast to the consistent strong enhancement of CD8^+^ activation observed across donors when cocultured with *MGAT1* KO iDCs. Because the baseline level of T cell activation varied between donors, we sought to enhance T cell stimulation and thereby reduce inter-donor variability. PD-L1 blockade disrupts the PD-1/PD-L1 interaction, relieving inhibitory signaling and enhancing T cell activation (51). PD-L1 inhibition increased activation across all donors and partially reduced donor-to-donor variability due to the higher activation baseline (**Figures 5E, F**). No significant differences between WT and *MGAT1* KO iDCs were observed among CD4^+^ T cells following aPD-L1 treatment. In contrast, *MGAT1* KO iDCs continued to induce markedly higher frequencies of activated and proliferating CD8^+^ T cells compared with WT, demonstrating that the *MGAT1* KO–driven enhancement of CD8^+^ T cell activation persists even under conditions of strongly increased overall T cell activation.

Interestingly, our previous study investigating *ST6GAL1* KO showed enhanced HLA-DR MFI, but not HLA-ABC. Additionally, *ST6GAL1* KO resulted in increased CD4^+^ T cell activation, but no CD8^+^ T cell activation compared to WT. Upon neuraminidase treatment of the *ST6GAL1* KO iDCs, increased CD8^+^ T cell activation was observed as well (25). These data, in combination with the consistent increased CD8^+^ T cell activation observed after stimulation with *MGAT1* KO iDC in this study, suggests that α2,3 sialic acids or α2,8 polysialylation (4, 52) on DC N-glycans either inhibit CD8^+^ T cell activation or that their removal results in increased activation. While removal of N-linked α2,6 sialic acids appear to consistently increase CD4^+^ T cell activation, as observed in our previous *ST6GAL1* KO study (25), a shift to oligomannose N-glycans and consequently a complete loss of sialylated hybrid and complex N-glycans does not increase the CD4^+^ T cell activation as profoundly. This points to a potential role for desialylated hybrid and complex N-glycans in facilitating enhanced CD4^+^ T cell activation or implies that additional modifications, such as alternative capping of N-glycans may be crucial for modulating this response. Taken together, these findings also demonstrate how the specific type of sialic acid linkages can lead to significantly different functional outcomes.

The increased CD8^+^ T cell activation was accompanied by an elevated mRNA level of *NFKB1* in *MGAT1* KO iDCs (**Figure 3C**). *NFKB1* encodes a DNA-binding subunit of the NF-κB complex, a master regulator of immune and inflammatory responses (**Figure 3C**) (53, 54). The NF-κB transcription factor is essential for DC maturation and the activation of NF-κB controls the expression of HLA-ABC/DR and key costimulatory molecules like CD40, along with various proinflammatory cytokines (54). Inhibition of NF-κB activity in DCs has been shown markedly inhibit T cell proliferation and reduce IL-2 and IFNγ production in a MLR (53, 55). The substantial increase of *NFKB1* mRNA in *MGAT1* KO iDCs strongly supports a direct association between enhanced NF-κB activity and the increased surface expression of CD40, HLA-ABC, and HLA-DR ultimately driving increased T cell proliferation and cytokine secretion (**Figures 3A, B**, **4B**). Additionally, *IFNB1* expression was significantly increased in *MGAT1* KO iDCs, with *IRF1* showing a similar trend, though not statistically significant (**Figure 3C**). IRF1 is a primary transcription factor regulating IFN-mediated gene expression, including type 1 interferon *IFNB1* (56, 57). The NF-κB and IFN pathways were recently described to be highly enriched in a certain functionally mature subpopulation of conventional type 1 dendritic cells (cDC1s) (57). A defining characteristic of cDC1s is their ability to cross-present antigens via MHC-I to CD8^+^ T cells, a process essential for effective antitumor immunity (17, 57). This functional capacity is closely linked to the activity of NF-κB and IRF1, as the inhibition of either transcription factor impairs CD8^+^ T cell activation (57). Consistent with previous studies highlighting the important roles of NF-κB and interferon related genes in DC function, our findings demonstrate that in *MGAT1* KO iDCs enhanced *NFKB1* expression correlates with increased surface expression of HLA-ABC, HLA-DR, and CD40. In coculture, this phenotype is further linked to enhanced CD8^+^ T cell activation and proliferation, accompanied by elevated production of IL-2 and IFNγ.

We also observed a significantly decreased expression of *SIGLEC7* in *MGAT1* KO iDCs compared to WT iDCs. *SIGLEC7* encodes the sialic acid–binding immunoglobulin-like lectin 7 (Siglec-7) which is an inhibitory glycan-binding immune receptor expressed on various immune cells, including DCs (58–60). Disialyl core-1 O-glycans are the primary immune ligands for Siglec-7 and are particularly abundant on naïve T cells. Blocking of Siglec-7 in DCs has been shown to enhance the activation of both primary T cells and antigen-presenting DCs *in vitro* (59). The lower *SIGLEC7* expression observed in *MGAT1* KO iDCs may attenuate tolerogenic signaling, thereby further enhancing T cell activation.

Altogether, the observed changes in genotype and phenotype in *MGAT1* KO iDCs provide a plausible explanation for their altered behavior and enhanced ability to prime CD8^+^ T cells, as our data highlights key differences in proinflammatory regulators and antigen-presenting molecules. While these findings offer valuable insights into the molecular and functional impact of *MGAT1* KO in human DCs, further studies are needed to fully elucidate the precise mechanisms underlying these effects.

Given the numerous N-glycosylated receptors on the DC surface, including key costimulatory molecules, it is possible that changes in glycosylation patterns impact receptor–ligand binding affinities with T cell receptors, thereby altering T cell activation. In addition, many cytokines and cytokine receptors are N-glycosylated and in *MGAT1* KO DCs, the secreted cytokines and receptors will present oligomannose N-glycans (61, 62). Glycosylation is known to influence receptor binding, stability, and the function of cytokines, offering a plausible mechanism for distinct effects in T cell activation observed between WT and *MGAT1* KO DCs (63). Additionally, cytokine receptors on *MGAT1* KO DCs might exhibit altered affinities for differentiation cytokines such as GM-CSF, TNF-α, and IL-4, thus impacting differentiation responses. All receptors binding these cytokines are N-glycosylated, and intact N-glycosylation sites are essential for high-affinity GM-CSF receptor binding and N-glycosylation has been found vital for TNFR1’s binding to TNFα and supports a TNFα autocrine loop, which amplifies inflammation through NF-κB pathways in microglia (64–66). Consistent with this, our data demonstrates that *MGAT1* KO MUTZ-3 precursor cells respond more rapidly to cytokine stimulation and acquire DC-associated surface markers such as CD1a and CD86 earlier during differentiation (**Figure 6B**). By day 3, *MGAT1* KO early iDCs already exhibit significantly elevated expressions of CD40, HLA-ABC, and HLA-DR compared to PC stage, indicating accelerated progression toward a DC-like phenotype (**Figure 6C**). Functionally, these early *MGAT1* KO iDCs were already capable of inducing strong T cell proliferation, whereas WT early iDCs displayed minimal stimulatory capacity (**Figures 6D, E**). Together, these findings suggest that loss of *MGAT1* and the resulting shift to oligomannose N-glycans enhance the responsiveness of DC precursors to cytokine signaling, promoting faster differentiation into immunocompetent DCs and contributing to the increased T cell activation observed in mature *MGAT1* KO iDCs.

The primary focus of the present study was to investigate how *MGAT1* impacts DC function and T cell activation, using allogeneic cocultures as the central approach. Utilizing this widely used and accepted experimental setup to investigate the overall immunostimulatory capacity of *MGAT1* KO DCs, our data demonstrates that a *MGAT1* KO accelerates the differentiation into DCs and that *MGAT1* KO DCs consistently enhance CD8^+^ T cell activation. However, additional studies are needed to determine whether *MGAT1* KO also affects antigen processing and presentation. While previous work has demonstrated that MUTZ-3 DCs successfully can be loaded with antigens and used to prime tumor-specific cytotoxic T cells (28), antigen-specific T cell activation assays and antigen uptake assays will be important to fully evaluate the potential of *MGAT1* KO DCs in the context of antigen processing and presentation. Our isogenic DC model provides a strong foundation for future studies investigating how altered glycosylation shapes antigen handling and the induction of antigen-specific immune responses.

Overall, this study reveals how *MGAT1* KO in DCs generates a markedly enhanced immunostimulatory profile compared to WT DCs. The KO significantly boosts CD8^+^ T cell activation and cytokine production, thereby offering a promising strategy to improve DC performance in immunotherapy. When considered alongside our previous findings showing that *ST6GAL1* KO selectively boosted CD4^+^ T cell activation (25), these results suggest that targeted glycoengineering can fine-tune immune responses and potentially steer activation toward specific T cell subsets. Overall, these findings highlight the potential of glycoengineering and glycooptimization to advance immune modulation for therapeutic applications.

## Data Availability

The original contributions presented in the study are included in the article/**Supplementary Material**, further inquiries can be directed to the corresponding author.

## References

[1] Fu C Zhou L Mi QS Jiang A . Dc-based vaccines for cancer immunotherapy. Vaccines. (2020) 8:1–16. doi: 10.3390/vaccines8040706, PMID: 33255895 PMC7712957

[2] He M Zhou X Wang X . Glycosylation: mechanisms, biological functions and clinical implications. Signal Transduction Targeted Ther. (2024) 9. doi: 10.1038/s41392-024-01886-1, PMID: 39098853 PMC11298558

[3] Wculek SK Cueto FJ Mujal AM Melero I Krummel MF Sancho D . Dendritic cells in cancer immunology and immunotherapy. Nat Rev Immunol. (2020) 20:7–24. doi: 10.1038/s41577-019-0210-z, PMID: 31467405

[4] Bax M García-Vallejo JJ Jang-Lee J North SJ Gilmartin TJ Hernandez G . Dendritic cell maturation results in pronounced changes in glycan expression affecting recognition by siglecs and galectins. J Immunol. (2007) 179:8216–24. doi: 10.4049/jimmunol.179.12.8216, PMID: 18056365

[5] Crespo HJ Guadalupe Cabral M Teixeira AV Lau JTY Trindade H Videira PA . Effect of sialic acid loss on dendritic cell maturation. Immunology. (2009) 128:e621–31. doi: 10.1111/j.1365-2567.2009.03047.x, PMID: 19740323 PMC2753891

[6] Videira PA Amado IF Crespo HJ Carmen Algueró M Dall F Guadalupe Cabral M . Surface α2-3-and α2-6-sialylation of human monocytes and derived dendritic cells and its influence on endocytosis. Glycoconjugate J. (2007) 25:259–68. doi: 10.1007/s10719-007-9092-6, PMID: 18080182

[7] Baum LG Cobb BA . The direct and indirect effects of glycans on immune function. Glycobiology. (2017) 27:619–24. doi: 10.1093/glycob/cwx036, PMID: 28460052

[8] Schjoldager KT Narimatsu Y Joshi HJ Clausen H . Global view of human protein glycosylation pathways and functions. Nat Rev Mol Cell Biol. (2020) 21:729–49. doi: 10.1038/s41580-020-00294-x, PMID: 33087899

[9] Schlickeiser S Stanojlovic S Appelt C Vogt K Vogel S Haase S . Control of TNF-induced dendritic cell maturation by hybrid-type N-glycans. J Immunol. (2011) 186:5201–11. doi: 10.4049/JIMMUNOL.1003410, PMID: 21422246

[10] Del Prete A Salvi V Soriani A Laffranchi M Sozio F Bosisio D . Dendritic cell subsets in cancer immunity and tumor antigen sensing. Cell Mol Immunol. (2023) 20:432–47. doi: 10.1038/s41423-023-00990-6, PMID: 36949244 PMC10203372

[11] Janssen LLG Westers TM Rovers J Valk PJM Cloos J De Gruijl TD . Durable responses and survival in high-risk myelodysplastic syndrome and acute myeloid leukemia patients receiving the allogeneic leukemia-derived dendritic cell vaccine DCP-001. HemaSphere. (2023) 7:E968. doi: 10.1097/HS9.0000000000000968, PMID: 37928626 PMC10624465

[12] Borges F Laureano RS Vanmeerbeek I Sprooten J Demeulenaere O Govaerts J . Trial watch: anticancer vaccination with dendritic cells. OncoImmunology. (2024) 13:2412876. doi: 10.1080/2162402X.2024.2412876, PMID: 39398476 PMC11469433

[13] Lee KW Yam JWP Mao X . Dendritic cell vaccines: A shift from conventional approach to new generations. Cells. (2023) 12:1–36. doi: 10.3390/cells12172147, PMID: 37681880 PMC10486560

[14] Saxena M Balan S Roudko V Bhardwaj N . Towards superior dendritic-cell vaccines for cancer therapy. Nat Biomed Eng. (2018) 2:341–4. doi: 10.1038/s41551-018-0250-x, PMID: 30116654 PMC6089533

[15] Acker HHV Versteven M Lichtenegger FS Roex G Campillo-Davo D Lion E . Dendritic cell-based immunotherapy of acute myeloid leukemia. J Clin Med. (2019) 8:579. doi: 10.3390/jcm8050579, PMID: 31035598 PMC6572115

[16] Elwakeel A Bridgewater HE Bennett J . Unlocking dendritic cell-based vaccine efficacy through genetic modulation—How soon is now? Genes. (2023) 14:2118. doi: 10.3390/genes14122118, PMID: 38136940 PMC10743214

[17] Laureano RS Sprooten J Vanmeerbeerk I Borras DM Govaerts J Naulaerts S . Trial watch: Dendritic cell (DC)-based immunotherapy for cancer. OncoImmunology. (2022) 11:1–19. doi: 10.1080/2162402X.2022.2096363, PMID: 35800158 PMC9255073

[18] Lutz MB Backer RA Clausen BE . Revisiting current concepts on the tolerogenicity of steady-state dendritic cell subsets and their maturation stages. J Immunol. (2021) 206:1681–9. doi: 10.4049/jimmunol.2001315, PMID: 33820829

[19] Hannani D Leplus E Laurin D Caulier B Aspord C Madelon N . A new plasmacytoid dendritic cell-based vaccine in combination with anti-PD-1 expands the tumor-specific CD8+ T cells of lung cancer patients. Int J Mol Sci. (2023) 24:1896. doi: 10.3390/ijms24031897, PMID: 36768214 PMC9915756

[20] Plumas J . Harnessing dendritic cells for innovative therapeutic cancer vaccines. Curr Opin Oncol. (2022) 34:161–8. doi: 10.1097/CCO.0000000000000815, PMID: 34930882

[21] Charles J Chaperot L Hannani D Bruder Costa J Templier I Trabelsi S . An innovative plasmacytoid dendritic cell line-based cancer vaccine primes and expands antitumor T-cells in melanoma patients in a first-in-human trial. OncoImmunology. (2020) 9:1–13. doi: 10.1080/2162402X.2020.1738812, PMID: 32313721 PMC7153838

[22] Lenogue K Walencik A Laulagnier K Molens JP Benlalam H Dreno B . Engineering a human plasmacytoid dendritic cell-based vaccine to prime and expand multispecific viral and tumor antigen-specific t-cells. Vaccines. (2021) 9:1–15. doi: 10.3390/vaccines9020141, PMID: 33578850 PMC7916617

[23] van de Loosdrecht AA van Wetering S Santegoets SJAM Singh SK Eeltink CM den Hartog Y . A novel allogeneic off-the-shelf dendritic cell vaccine for post-remission treatment of elderly patients with acute myeloid leukemia. Cancer Immunology Immunotherapy. (2018) 67:1505–18. doi: 10.1007/s00262-018-2198-9, PMID: 30039426 PMC6182404

[24] van de Loosdrecht AA Wagner Drouet E Platzbecker U Holderried TAW Van Elssen C Giagounidis A . Induction of cellular and humoral immune responses is associated with durable remissions in MRD+ AML-patients after maintenance treatment with an allogeneic leukemia-derived dendritic cell vaccine. Blood. (2023) 142:769. doi: 10.1182/blood-2023-185532 37339577

[25] Tian W Blomberg AL Steinberg KE Henriksen BL Jørgensen JS Skovgaard K . Novel genetically glycoengineered human dendritic cell model reveals regulatory roles of α2,6-linked sialic acids in DC activation of CD4 + T cells and response to TNFα. Glycobiology. (2024) 34:cwae042. doi: 10.1093/glycob/cwae042, PMID: 38873803

[26] Goletz S Scheper RJ Masterson AJ Pinedo HM . *Differentiation of MUTZ-3 cells to produce effective dendritic cells* (Patent US8455253B2). Washington, D.C.: U.S. Patent and Trademark Office (USPTO) (2001).

[27] Larsson K Lindstedt M Borrebaeck CAK . Functional and transcriptional profiling of MUTZ-3, a myeloid cell line acting as a model for dendritic cells. Immunology. (2006) 117:156–66. doi: 10.1111/J.1365-2567.2005.02274.X, PMID: 16423051 PMC1782214

[28] Masterson AJ Sombroek CC De Gruijl TD Graus YMF van der Vliet HJJ Lougheed SM . MUTZ-3, a human cell line model for the cytokine-induced differentiation of dendritic cells from CD34+ precursors. Blood. (2002) 100:701–3. doi: 10.1182/blood.V100.2.701, PMID: 12091369

[29] Ruben JM Visser LL Heinhuis KM O’Toole T Bontkes HJ Westers TM . A human cell line model for interferon-α Driven dendritic cell differentiation. PloS One. (2015) 10:e0135219. doi: 10.1371/journal.pone.0135219, PMID: 26252775 PMC4529224

[30] Santegoets SJAM Marco AE Schreurs WJ Masterson AJ Poi Y Ae L . *In vitro* priming of tumor-specific cytotoxic T lymphocytes using allogeneic dendritic cells derived from the human MUTZ-3 cell line. Cancer Immunol Immunother. (2005) 55:1480–90. doi: 10.1007/s00262-006-0142-x, PMID: 16468034 PMC11030798

[31] Santegoets SJAM Masterson AJ van der Sluis PC Lougheed SM Fluitsma DM van den Eertwegh AJM . A CD34 + human cell line model of myeloid dendritic cell differentiation: evidence for a CD14 + CD11b + Langerhans cell precursor. J Leukocyte Biol. (2006) 80:1337–44. doi: 10.1189/JLB.0206111, PMID: 16959899

[32] Santegoets SJAM van den Eertwegh AJM van de Loosdrecht AA Scheper RJ de Gruijl TD . Human dendritic cell line models for DC differentiation and clinical DC vaccination studies. J Leukocyte Biol. (2008) 84:1364–73. doi: 10.1189/jlb.0208092, PMID: 18664532

[33] Balneger N Cornelissen LAM Wassink M Moons SJ Boltje TJ Bar-Ephraim YE . Sialic acid blockade in dendritic cells enhances CD8+ T cell responses by facilitating high-avidity interactions. Cell Mol Life Sci. (2022) 79:98. doi: 10.1007/S00018-021-04027-X, PMID: 35089436 PMC8799591

[34] Edgar LJ Thompson AJ Vartabedian VF Kikuchi C Woehl JL Teijaro JR . Sialic acid ligands of CD28 suppress costimulation of T cells. ACS Cent Sci. (2021) 7:1508–15. doi: 10.1021/acscentsci.1c00525, PMID: 34584952 PMC8461770

[35] Jenner J Kerst G Handgretinger R Müller I . Increased α2,6-sialylation of surface proteins on tolerogenic, immature dendritic cells and regulatory T cells. Exp Hematol. (2006) 34:1211–7. doi: 10.1016/J.EXPHEM.2006.04.016, PMID: 16939814

[36] Stanley P Moremen KW Lewis NE Taniguchi N Aebi M . N-glycans. In: Essentials of glycobiology, fourth edition. Cold Spring Harbor (NY): Cold Spring Harbor Laboratory Press (2022).

[37] Narimatsu Y Joshi HJ Yang Z Gomes C Chen YH Lorenzetti FC . A validated gRNA library for CRISPR/Cas9 targeting of the human glycosyltransferase genome. Glycobiology. (2018) 28:295–305. doi: 10.1093/glycob/cwx101, PMID: 29315387

[38] Andersen CL Jensen JL Ørntoft TF . Normalization of real-time quantitative reverse transcription-PCR data: a model-based variance estimation approach to identify genes suited for normalization, applied to bladder and colon cancer data sets. Cancer Res. (2004) 64:5245–50. doi: 10.1158/0008-5472.CAN-04-0496, PMID: 15289330

[39] Vandesompele J De Preter K Pattyn F Poppe B Van Roy N De Paepe A . Accurate normalization of real-time quantitative RT-PCR data by geometric averaging of multiple internal control genes. Genome Biol. (2002) 3:research0034.1. doi: 10.1186/gb-2002-3-7-research0034, PMID: 12184808 PMC126239

[40] Madsen AV Mejias-Gomez O Pedersen LE Skovgaard K Kristensen P Goletz S . Immobilization-free binding and affinity characterization of higher order bispecific antibody complexes using size-based microfluidics. (2022) 94:13652–58. doi: 10.1021/acs.analchem.2c02705, PMID: 36166291 PMC9558742

[41] Madsen AV Kristensen P Buell AK Goletz S . Generation of robust bispecific antibodies through fusion of single-domain antibodies on IgG scaffolds: a comprehensive comparison of formats. (2023) 15:2189432. doi: 10.1080/19420862.2023.2189432, PMID: 36939220 PMC10038023

[42] Bojar D Meche L Meng G Eng W Smith DF Cummings RD . A useful guide to lectin binding: machine-learning directed annotation of 57 unique lectin specificities. ACS Chem Biol. (2022) 17:2993–3012. doi: 10.1021/acschembio.1c00689, PMID: 35084820 PMC9679999

[43] Varki A Cummings RD Aebi M Packer NH Seeberger PH Esko JD Kornfeld S . Symbol nomenclature for graphical representations of glycans. Glycobiology. (2015) 25:1323–4. doi: 10.1093/glycob/cwv091, PMID: 26543186 PMC4643639

[44] Annunziato F Romagnani C Romagnani S . The 3 major types of innate and adaptive cell-mediated effector immunity. J Allergy Clin Immunol. (2015) 135:626–35. doi: 10.1016/j.jaci.2014.11.001, PMID: 25528359

[45] Shimabukuro-Vornhagen A Draube A Liebig TM Rothe A Kochanek M Von Bergwelt-Baildon MS . The immunosuppressive factors IL-10, TGF-β, and VEGF do not affect the antigen-presenting function of CD40-activated B cells. J Exp Clin Cancer Res. (2012) 31:1–7. doi: 10.1186/1756-9966-31-47, PMID: 22592077 PMC3443023

[46] Hoek M Demmers LC Wu W Heck AJR . Allotype-specific glycosylation and cellular localization of human leukocyte antigen class i proteins. J Proteome Res. (2021) 20:4518–28. doi: 10.1021/acs.jproteome.1c00466, PMID: 34415762 PMC8419865

[47] Ryan SO Cobb BA . Roles for major histocompatibility complex glycosylation in immune function. Semin Immunopathology. (2012) 34:425–41. doi: 10.1007/s00281-012-0309-9, PMID: 22461020 PMC3884644

[48] Pereira MS Alves I Vicente M Campar A Silva MC Padrão NA . Glycans as key checkpoints of T cell activity and function. Front Immunol. (2018) 9:2754. doi: 10.3389/fimmu.2018.02754, PMID: 30538706 PMC6277680

[49] Silva Z Ferro T Almeida D Soares H Ferreira JA Deschepper FM . MHC class I stability is modulated by cell surface sialylation in human dendritic cells. Pharmaceutics. (2020) 12:249. doi: 10.3390/pharmaceutics12030249, PMID: 32164343 PMC7150992

[50] St Paul M Ohashi PS . The roles of CD8 + T cell subsets in antitumor immunity. Trends Cell Biol. (2020) 30:695–704. doi: 10.1016/j.tcb.2020.06.003, PMID: 32624246

[51] Lee HT Lee JY Lim H Lee SH Moon YJ Pyo HJ . Molecular mechanism of PD-1/PD-L1 blockade via anti-PD-L1 antibodies atezolizumab and durvalumab. Sci Rep. (2017) 7:5532. doi: 10.1038/s41598-017-06002-8, PMID: 28717238 PMC5514103

[52] Kiermaier E Moussion C Veldkamp CT Gerardy-Schahn R De Vries I Williams LG . Polysialylation controls dendritic cell trafficking by regulating chemokine recognition. Science. (2016) 351:186–90. doi: 10.1126/science.aad0512, PMID: 26657283 PMC5583642

[53] Tas SW de Jong EC Hajji N May MJ Ghosh S Vervoordeldonk MJ . Selective inhibition of NF-kappaB in dendritic cells by the NEMO-binding domain peptide blocks maturation and prevents T cell proliferation and polarization. Eur J Immunol. (2005) 35:1164–74. doi: 10.1002/EJI.200425956, PMID: 15770694

[54] Yoshimura S Bondeson J Foxwell BMJ Brennan FM Feldmann M . Effective antigen presentation by dendritic cells is NF-κB dependent: Coordinate regulation of MHC, co-stimulatory molecules and cytokines. Int Immunol. (2001) 13:675–83. doi: 10.1093/intimm/13.5.675, PMID: 11312255

[55] Hernandez A Burger M Blomberg BB Ross WA Gaynor JJ Lindner I . Inhibition of NF-κB during human dendritic cell differentiation generates anergy and regulatory T-cell activity for one but not two human leukocyte antigen DR mismatches. Hum Immunol. (2007) 68:715–29. doi: 10.1016/j.humimm.2007.05.010, PMID: 17869645 PMC2245875

[56] Feng H Zhang YB Gui JF Lemon SM Yamane D . Interferon regulatory factor 1 (IRF1) and anti-pathogen innate immune responses. PloS Pathog. (2021) 17:1–22. doi: 10.1371/journal.ppat.1009220, PMID: 33476326 PMC7819612

[57] Ghislat G Cheema A Baudoin E Verthuy C Ballester PJ Crozat K . NF-kB–dependent IRF1 activation programs cDC1 dendritic cells to drive antitumor immunity. Sci Immunol. (2021) 6:eabg3570. doi: 10.1126/sciimmunol.abg3570, PMID: 34244313

[58] Boelaars K van Kooyk Y . Targeting myeloid cells for cancer immunotherapy: Siglec-7/9/10/15 and their ligands. Trends Cancer. (2024) 10:230–41. doi: 10.1016/j.trecan.2023.11.009, PMID: 38160071

[59] Stewart N Daly J Drummond-Guy O Krishnamoorthy V Stark JC Riley NM . The glycoimmune checkpoint receptor Siglec-7 interacts with T-cell ligands and regulates T-cell activation. J Biol Chem. (2024) 300:105579. doi: 10.1016/j.jbc.2023.105579, PMID: 38141764 PMC10831161

[60] Wang J Manni M Bärenwaldt A Wieboldt R Kirchhammer N Ivanek R . Siglec receptors modulate dendritic cell activation and antigen presentation to T cells in cancer. Front Cell Dev Biol. (2022) 10:828916. doi: 10.3389/fcell.2022.828916, PMID: 35309936 PMC8927547

[61] Scapin G Cagdas E Marie L Lewis NE Goletz S Hafkenscheid L . Implications of glycosylation for the development of selected cytokines and their derivatives for medical use. Biotechnol Adv. (2024) 77:108467. doi: 10.1016/j.bioteChadv.2024.108467, PMID: 39447666 PMC12228884

[62] Sun R Min A Kim J Lim S . Glycosylation of immune receptors in cancer. Cells. (2021) 10:1100. doi: 10.3390/cells10051100, PMID: 34064396 PMC8147841

[63] Anderluh M Berti F Bzducha-Wróbel A Chiodo F Colombo C Compostella F . Emerging glyco-based strategies to steer immune responses. FEBS J. (2021) 288:4746–72. doi: 10.1111/febs.15830, PMID: 33752265 PMC8453523

[64] Kim J Jung K Kim J Kim S Park H Kim Y . Engineering of anti-human interleukin-4 receptor alpha antibodies with potent antagonistic activity. Sci Rep. (2019) 9:7772. doi: 10.1038/s41598-019-44253-9, PMID: 31123339 PMC6533264

[65] Niu L Heaney ML Vera JC Golde DW . High-affinity binding to the GM-CSF receptor requires intact N- glycosylation sites in the extracellular domain of the β subunit. Blood. (2000) 95:3357–62. doi: 10.1182/blood.v95.11.3357, PMID: 10828016

[66] Yu J Sun H Cao W Song Y Jiang Z . Research progress on dendritic cell vaccines in cancer immunotherapy. Exp Hematol Oncol. (2022) 11:1–22. doi: 10.1186/s40164-022-00257-2, PMID: 35074008 PMC8784280

