# Supplementary material for: MGAT1 knockout in human dendritic cells enhance CD8+ T cell activation
Source: Front Immunol. 2025 Dec 17;16:1588795. doi: 10.3389/fimmu.2025.1588795 (PMC12753448; doi:10.3389/fimmu.2025.1588795)
Supplement: Supplementary file 1 [file Table1.docx]

**Supplementary Material**

| **Lectin** | **Company** | **Catalog #** | **Working concentration  (µg/mL)** |
| --- | --- | --- | --- |
| Aleuria Aurantia Lectin (AAL) | Vector Laboratories | B-1395 | 0.078 |
| Maackia Amurensis Lectin I (MAL-I) | Vector Laboratories | B-1315 | 0.15 |
| Maackia Amurensis Lectin II (MAL-II) | Vector Laboratories | B-1265 | 0.15 |
| Phaseolus Vulgaris Leucoagglutinin (PHA-L) | Vector Laboratories | B-1115 | 0.15 |
| Sambucus Nigra Lectin (SNA) | Vector Laboratories | B-1305 | 0.15 |
| Anti-Peanut Agglutinin (PNA) | Vector Laboratories | B-1075 | 0.5 |
|  |  |  |  |

Table S1: Lectins used for cell surface glycoprofiling
