# Supplementary material for: MGAT1 knockout in human dendritic cells enhance CD8+ T cell activation
Source: Front Immunol. 2025 Dec 17;16:1588795. doi: 10.3389/fimmu.2025.1588795 (PMC12753448; doi:10.3389/fimmu.2025.1588795)
Supplement: Supplementary file 2 [file Table2.docx]

| **Gene name** | **Forward primer** | **Reverse primer** |
| --- | --- | --- |
| *CCL3*  *CCR7*  *CD14*  *CD1A*  *CD209*  *CD274*  *CD34*  *CD40*  *CD58*  *CD70*  *CD83*  *CD86*  *CLEC2B*  *CXCL8*  *CXCR1*  *CXCR2*  *GAPDH*  *HLA-A*  *HLA-B*  *HLA-C*  *HLA-DPA1*  *HLA-DQA1*  *HLA-DRA*  *HLA-E*  *ICAM*  *IFNB1*  *IL1A*  *IL1B*  *IL23A*  *IRF1*  *IRF4*  *ITGAM*  *ITGAX*  *MYD88*  *NFKB1*  *RPLP0*  *SIGLEC1*  *SIGLEC10*  *SIGLEC15*  *SIGLEC7*  *SIGLEC8*  *SIGLEC9*  *STAT3*  *STAT5B*  *TLR2*  *TLR6*  *TNF*  *TNFAIP6*  *TNFRSF1A* | CCGGTGTCATCTTCCTAACC  CTCTTCCTCCTGACCCTTCC  TTGTGAGCTGGACGATGAAG  AGCTGTTCTCCCAGGTGATG  ACAGAGGAGAGCCCAACAAC  GCTGAATTGGTCATCCCAGA  GCACCAATCTGACCTGAAAAA  GTGGTGATCCCCATCATCTT  GCGGTCATTCAAGACACAGA  GTCACTTGGGTGGGACGTAG  CAACTCGGGGACATACAGGT  AAGTATATGGGCCGCACAAG  TGGACTGAAGATGGCAAAAA  AGCTCTGTGTGAAGGTGCAG  GCAGCTCCTACTGTTGGACA  TGGAGGTGTCCTACAGGTGA  GAGTCAACGGATTTGGTCGT  TGGAGGAGGAAGAGCTCAGA  TCCGCAGATACCTGGAGAAC  CAGAACCCCCAAAGACACAC  GACCTTCCAGATCCTGGTGA  CCAACTCTACCGCTGCTACC  AGCACTGGGAGTTTGATGCT  CTGGAGAAGGGGAAGGAGAC  GAACCAGAGCCAGGAGACAC  TTCACCAGGGGAAAACTCAT  CAGCCAGAGAGGGAGTCATT  AGCTGATGGCCCTAAACAGA  AGCTTCATGCCTCCCTACTG  AGAGCCAACATGCCCATC  GGACTACAACCGCGAGGAG  GCCGGTGAAATATGCTGTCT  GTCTACCACCAGCCCTTCC  CTCTCTCCTTCCCAGAGCAA  CATATTTGGGAAGGCCTGAA  GCGACCTGGAAGTCCAACTA  GCGTGTTTGTAAGCAGAGCA  CCGAAGAGACGGACTCAGAC  CTTCAAGGCGCTGCTGCT  GGAAGGATTACTCGCTGACG  TCGGCTAGAGAGAGGAAGCA  CAGAGCTCAGAACCCTCTCG  GTCCTGAGCTGGCAGTTCTC  CCATGGCTGTGTGGATACAA  CCTGGGCAGTCTTGAACATT  AGTTCTCCGACGGAAATGAA  CTCTTCTGCCTGCTGCACTT  GCTAGAGGCAGCCAGAAAAA  CCCTCAGGGGTTATTGGACT | TTTCTGGACCCACTCCTCAC  AGCTTGCAAAAGTGGACACC  TGCAGACACACACTGGAAGG  TAAAAGGATGCGATCCAGGT  CAGATCCAGAATTTGGCAAGA  TGGCTCCCAGAATTACCAAG  GAATAGCTCTGGTGGCTTGC  CTTCTTGGCCACCTTTTTGA  TGTCACATTTCAGAATACCATTCA  CCAGTATAGCCTGGGGTCCT  AGGGCATCCTGTCACTCTCA  GCCCTTGTCCTTGATCTGAA  TGCCAAACGATTTGGTAAATG  AAATTTGGGGTGGAAAGGTT  CCTCTTCAGTTTCAGCAATGG  TCTTCAAAGCTGTCACTCTCCA  AATGAAGGGGTCATTGATGG  GCTGTGAGGGACACATCAGA  GTGGGTCACGTGTGTCTTTG  GAAGGTTCCATCTCCTGCTG  ACTATCCAGGCTGGTGTGCT  CCACAAGACAGATGAGGGTGT  ATAATGATGCCCACCAGACC  GTGGCCTCATGGTCAGAGAT  CCTCTGGCTTCGTCAGAATC  TCCTTGGCCTTCAGGTAATG  GTCTGGAACTTTGGCCATCT  GGAGATTCGTAGCTGGATGC  GACTGAGGCTTGGAATCTGC  GGGATTTGGTTGGAATTAATCTG  GCCTTCTCGGAACTTTCCTT  TTCTCTGAGGCCGTGAAGTT  GAGCTCCTCAAAGCCTTGGT  CACCTAAGACCATGGCACCT  CAGTGCCATCTGTGGTTGAA  TGTCTGCTCCCACAATGAAA  GCTGCATCAGGATCAATGAG  TCGGGACCACATTGATGTAA  CTTCAAGGCGCTGCTGCT  GCTGTCCACTGGGTAGGAGA  TGGATGCTGTCACAAACACA  CCCCTGAGTCACTCCTGATG  CACACCAGGTCCCAAGAGTT  ACCTCAATGGGAAAATGCTG  AGAAGAAAGGGGCTTGAACC  GCGGTAGGTCTTTTGGAACA  GCCAGAGGGCTGATTAGAGA  CTGGCTTCACAATGGGGTAT  ATTTTCCTTGGGGACACACA |

Table S2: Primers used for RT-qPCR
