# Supplementary material for: MGAT1 knockout in human dendritic cells enhance CD8+ T cell activation
Source: Front Immunol. 2025 Dec 17;16:1588795. doi: 10.3389/fimmu.2025.1588795 (PMC12753448; doi:10.3389/fimmu.2025.1588795)
Supplement: Supplementary file 3 [file Table3.docx]

| **Antibody** | **Company** | **Catalog#** | **Dilution** |
| --- | --- | --- | --- |
| HLA-DR-BV650 | BD Biosciences | 564231 | 1/100 |
| CD209-PE | Biolegend | 330106 | 1/100 |
| CCR6-BV650 | Biolegend | 353426 | 1/100 |
| HLA-ABC-APC | eBioscience | 17-9983-42 | 1/100 |
| CD80-PerCP-eFluor 710 | eBioscience | 46-0809-42 | 1/100 |
| CD274-APC | eBioscience | 17-5983-42 | 1/100 |
| CD86-APC-Vio770 | Miltenyi | 130-116-163 | 1/100 |
| CD40-PE-Vio770 | Miltenyi | 130-110-948 | 1/100 |
| CD34-APC-Vio770 | Miltenyi | 130-124-457 | 1/100 |
| CD14-VioBlue | Miltenyi | 130-110-524 | 1/100 |
| CD8-APC-Vio770 | Miltenyi | 130-110-681 | 1/100 |
| CD4-VioBright 667 | Miltenyi | 130-114-532 | 1/100 |
| CD25-PE | Miltenyi | 130-113-286 | 1/100 |
| CD1a-APC-Vio770 | Miltenyi | 130-111-873 | 1/100 |
| CD3-PerCP-Vio700 | Miltenyi | 130-113-141 | 1/100 |

Table S3: Antibodies used for phenotyping.
